# The influence of employment social support for risk and prognosis in nonspecific back pain: a systematic review and critical synthesis

**DOI:** 10.1007/s00420-012-0804-2

**Published:** 2012-08-09

**Authors:** Paul Campbell, Gwenllian Wynne-Jones, Sara Muller, Kate M. Dunn

**Affiliations:** Arthritis Research UK Primary Care Centre, Primary Care Sciences, Keele University, Keele, Staffordshire, ST5 5BG UK

**Keywords:** Work social support, Social network, Back pain, Systematic review, Employment

## Abstract

**Purpose:**

To examine the influence of employment social support type (e.g. co-worker, supervisor, general support) on risk of occurrence of low back pain, and prognosis (e.g. recovery, return to work status) for those who have low back pain.

**Methods:**

Systematic search of seven databases (MEDLINE, Embase, PsychINFO, CINAHL, IBSS, AMED and BNI) for prospective or case–control studies reporting findings on employment social support in populations with nonspecific back pain. Data extraction and quality assessment were carried out on included studies. A systematic critical synthesis was carried out on extracted data.

**Results:**

Thirty-two articles were included that describe 46 findings on the effect of employment social support on risk of and prognosis of back pain. Findings show that there is no effect of co-worker, supervisor or general work support on risk of new onset back pain. Weak effects of employment support were found for recovery and return to work outcomes; greater levels of co-worker support and general work support were found to be associated with less time to recovery or return to work.

**Conclusions:**

The evidence suggests that the association between employment support and prognosis may be subject to influence from wider concepts related to the employment context. This review discusses these wider issues and offers directions for future research.

## Introduction

Nonspecific low back pain (LBP) is very common. Two large population studies (Papageorgiou et al. 1995; Cote et al. 1998) place a lifetime prevalence of back pain at 60–80 %. This high prevalence has considerable impact within the employment sector. For example, in a study of back pain consulters from a UK primary care sample (Wynne-Jones et al. 2008), 37 % of those unemployed attributed this to their back pain, 22 % of those currently employed were on sickness absence and a further 11 % were on reduced duties at work due to their back pain. A recent report by the European Work Foundation ‘*Fit for work*’ (Bevan et al. 2009) reports that 25 % of workers in Europe suffer from back pain and estimate the total cost of musculoskeletal illness on employment productivity in Europe at €12 billion. This is further compounded by evidence that the longer a person is out of work due to back pain, the more difficult it is to re-engage into employment, and that recurrence rates are high (Waddell and Burton 2001).

In the light of the impact of back pain on employment, there has been a steady growth in interest in what employment factors impact on both risk for back pain and related outcomes such as sickness absence, recovery and return to work (Hartvigsen et al. 2004; Steenstra et al. 2005). One influential theoretical model, utilised within employment and illness research, is Karasek’s Demand Control Model (Karasek et al. 1998). According to the model having a job with high demands (e.g. high paced physical work), with no or little control over the decisions affecting work (e.g. fixed schedules, having a subordinate position), leads to an increase in stress and subsequent illness (Landsbergis et al. 2001). It is proposed that these outcomes can be modified if the person receives social support within the employment context (Johnson and Hall 1988; Theorell and Karasek 1996). This and similar theoretical models have been investigated within musculoskeletal research (Bongers et al. 2006) and have led to clinical guidelines on the consideration of work psychosocial factors (Costa-Black et al. 2010).

However, the evidence within systematic reviews on the impact of employment social support on back pain has been conflicting. The reasons given for inconsistency include difficulties synthesising evidence, due to variation in the measurement of work social support (Bongers et al. 2006; Hartvigsen et al. 2004; Steenstra et al. 2005; Woods 2005), and a lack of research focus specifically on work social support; for example, of the eight recent reviews (Bongers et al. 2006; Hartvigsen et al. 2004; Steenstra et al. 2005; Woods 2005; Waddell and Burton 2001; Hoogendoorn et al. 2000; Kuijer et al. 2006; Lakke et al. 2009), only one review (Woods 2005) solely considered work support issues using qualitative methodology.

The objective of this systematic review is to describe the evidence of employment-related social support on the risk of occurrence of a new episode of back pain and on the influence of employment-related support on prognosis once someone has back pain (e.g. recovery, return to work status). Furthermore, by way of a critical evidence synthesis, this review will address some current difficulties reported by previous reviews. This will be done by (1) stratification of evidence by study outcome (e.g. risk or prognosis), (2) stratification by type of support (e.g. co-worker, supervisor, general support), (3) critical assessment of the evidence based on the adequacy of the measure of employment social support and other key components of the included studies (e.g. response rate, attrition rate, geographic location, type of employment, sample size, sophistication of the analysis, length of follow up time, assessment of LBP).

## Methods

This review uses a systematic approach to identify and synthesise research on employment social support (e.g. general level of support at work, level of supervisor support, level of co-worker support) within back pain populations.

### Search strategy

The following computerised databases were searched from their respective inception dates up to 18 November 2011: MEDLINE, Embase, PsychINFO, CINAHL, IBSS, AMED and BNI. Reference lists of the studies and current relevant reviews were checked for additional study citations. Validated measures of social support were also citation checked using the ISI Web of Science citation mapping system, and databases of local experts were consulted for information on additional research studies.

### Inclusion criteria

Articles were included if they had a focus on LBP populations (e.g. search term keywords: Back Pain, Low Back Pain), measured employment social support (e.g. search term keywords: Social Support, Social Interaction, Occupational Health Services, Employment Support, Employment Based Support), and provided data for the role of employment social support on risk of occurrence of LBP or prognosis with LBP outcomes such as pain intensity, disability or associated prognostic factors (search term keywords: Risk factors, Prospective, Epidemiologic Studies, Cohort studies, Case–Control Studies). The search terms (“Appendix 1”) were used as key words and also exploded to include all lower level headings (e.g. Mesh terms within MEDLINE).

### Exclusion criteria

Studies were excluded that focused exclusively on family support or informal social support or included populations with other specific health problems (e.g. cancer, diabetes), studies solely on pregnant women, studies of surgical cohorts (e.g. lumbar fusion patients), studies of back pain patients who have a specific diagnosis (e.g. lumbar stenosis, spondylolithesis, spinal cord diseases, red flags). Cross-sectional findings were also excluded due to the inability to distinguish cause and effect, as were small case series studies due to being underpowered (e.g. studies of <30 people).

### Procedure

Study abstracts were screened for clearly irrelevant studies, and for any study that was suitable, full text papers were obtained. Final selection of research papers was conducted by two reviewers (PC and KMD) using the inclusion and exclusion criteria.

### Assessment of study biases

All included articles were subject to quality assessment of study methodology for bias; the studies’ focus on employment social support, the measurement of social support, study population, analysis undertaken, and the quality of reporting. Further assessments were carried out relating to the study design type, such as the attrition rate and follow-up period as additional criteria for cohort studies or screening of controls within a case–control study designs. It was not possible to use a pre-existing quality assessment tool due to the inclusion of differing study designs (e.g. cohort, case control) and inclusion of specific assessments (i.e. social support, back pain) so the quality assessment measure (“Appendix 2”) was based on the combination of assessments of a number of recent review articles and guidance on quality assessment within systematic reviews on the area of back pain (Woods 2005; Kuijer et al. 2006; Mallen et al. 2007; Hayden et al. 2009).

Articles were assessed using the quality assessment criteria checklist by two reviewers (PC, GWJ). Thereafter, all disagreements were discussed at a consensus meeting, and if disagreements were not resolved, a third reviewer (KMD) provided the final judgement.

### Data extraction and synthesis

Study information on author, country, study population, sample size, response rate, follow-up period (cohort designs only), study design, focus, assessment of back pain, assessment of employment social support, analysis, outcome in relation to employment social support, findings and strength of reported effect were extracted from the studies. Full data extraction tables can be found in “Appendix 3”.

### Analysis

Studies were grouped together corresponding to their respective study design, occurrence (e.g. risk of back pain) and prognosis (e.g. disability, return to work, sickness absence, recovery). Studies were also grouped to reflect the type of employment social support reported within the research papers (e.g. co-worker support, supervisor support, unspecified work support). Studies that did not describe the specific type of support (i.e. unspecified work support) are described as ‘General Work Support’ (GWS) within this review. In addition, some studies had multiple outcomes within the analysis (e.g. a prospective cohort study reports on incident risk and follows up on disability or a study that report’s findings both on co-worker support and supervisor support) and were included within the findings more than once.

Studies were then stratified dependent on whether or not they reported a significant association of employment support on risk outcome (i.e. risk of LBP) or prognosis (i.e. sickness absence, return to work status). The analysis centred on comparisons between studies that reported an association or not using key aspects of extracted data, measurement of social support (studies that used a measure that included multiple items to assess support type were judged as adequate, studies that used a single item or did not specify were judged as poor), geographic location (countries where studies were carried out), worker sample (e.g. industrial workers, office workers, general workers), analysis type (e.g. univariate, multivariate), assessment of back pain (e.g. pain intensity, disability, mechanical assessment, medical codes, prevalence and duration), factors of study bias (sample size, baseline response, attrition, length of follow-up).

Assessment of strength of association was carried out following criteria guidelines (Hartvigsen et al. 2004; Iles et al. 2008); individual study results are described as: none (e.g. non-significant result), weak (e.g. OR/RR 1.01–1.49), moderate (e.g. OR/RR 1.50–1.99) or strong (e.g. OR/RR ≥ 2.0) in the support of an association between employment social support and back pain outcomes.

## Results

**Fig. 1:**
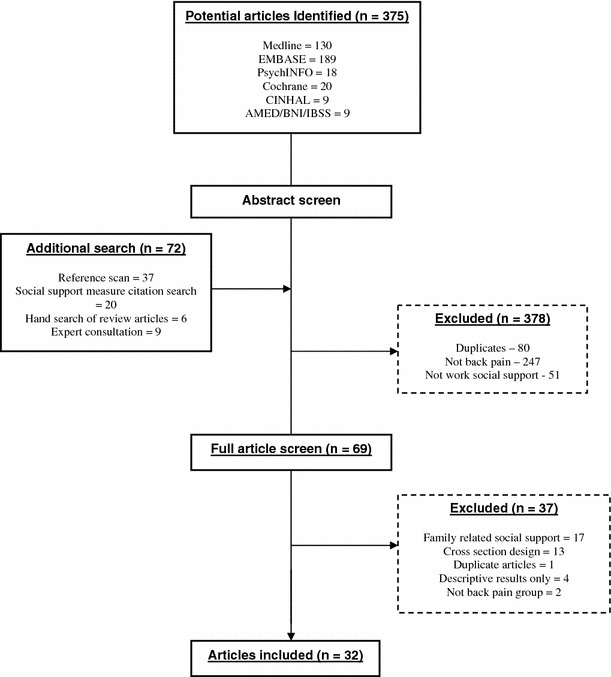
Flow diagram of review procedure

Systematic searching identified 375 publications (see Fig. 1). An additional 72 articles were included via alternative search strategies (hand search, expert consultation, and citation search). 378 articles were excluded following abstract screening (e.g. not nonspecific LBP population, duplicates) with a further 37 articles excluded following full text screening. The reasons for exclusion at the full text screening stage were studies solely focusing on family support, cross-sectional studies, studies on specific spinal pain populations (e.g. spondylolithesis, lumbar stenosis, spinal injury), or populations that focused on chronic pain patients outside of this study’s inclusion criteria (e.g. migraines, fibromyalgia, chronic widespread pain). This resulted in 32 suitable articles included within the review.

### Quality assessment analysis

Taken together, all studies offered a clear research objective, 91 % described their recruitment procedure adequately, 69 % described the demographics of their study populations and 56 % reached a quality target criteria of a 70 % participation rate (see “Appendix 2” for quality assessment scores for each study). Most (81 %) of the studies employed a citable measure of employment social support. For cohort designs, only 48 % of studies reported their attrition rates or reported comparisons of responders and non-responders and over half of the cohort studies reported a higher than 20 % attrition rate. Table 1 outlines the findings of employment social support for risk and prognosis for the included studies.

**Table 1:** Outcomes of low levels of employment social support on risk and prognosis for back pain

Outcome	Study	Study quality (%)	Strong support	Moderate support	Weak support	No support
Risk of occurrence for back pain	Andersen et al.	100				× (SS, CWS)
	Clays et al.	79			+ (GWS males)	× (GWS females)
	Elfering et al.	64				× (GWS)
	Feuerstein et al.	85			+ (SS)	
	Fransen et al.	50				× (GWS)
	Ghaffari et al.	64				× (GWS)
	Gheldof et al.	86				× (GWS)
	Gonge et al.	79				× (GWS)
	Harkness et al.	64				× (GWS)
	Hoogendoorn et al.	71				× (CWS, SS)
	Ijzelenberg and Burdorf	79	+ (SS)			× (CWS)
	Josephson and Vingard	78				× (GWS)
	Kaila-Kangas et al.	64	+ (SS)			× (CWS)
	Kerr et al.	92		− (CWS)		
	Krause et al.	86				× (CWS, SS)
	Larsman and Hanse	64				× (GWS)
	Leino and Hanninen	71		+ (GWS)		
	Rugulies and Krause	93				× (CWS, SS)
	Shannon et al.	79				× (GWS)
	Stevenson et al.	50	+ (CWS)			
Return to work/recovery	Dionne et al.	93				× (GWS)
	Gheldof et al.	86				× (GWS)
	Helmhout et al.	79				× (CWS, SS)
	Heymans et al.	86			+ (GWS)	
	Karlsson et al.	79				× (GWS)
	Lotters and Burdorf	71				× (GWS)
	Mielenz et al.	78		+ (CWS)		× (SS)
	Morken et al.	78			+ (GWS short term absence)	× (GWS long term absence)
	Schultz et al.	86		− (CWS)		
	Soucy et al.	79			+ (GWS)	
	Tubach et al.	86	+ (GWS, long term absence)			× (GWS, short term absence)
	van der Giezen et al.	79			+ (GWS)	
	van den Heuvel et al.	79	+ (CWS)			× (SS)

*LBP* Low back pain, *SS* supervisor support, *CWS* Co-worker support, *GWS* General work support, + positive association, − negative association, × (no association)

### Employment social support and risk of occurrence of back pain

In total, 20 studies report on 27 findings on the association of employment social support and occurrence of back pain. Of those findings, 20 reported no significant associations, one reported a strong reverse effect (a greater level of employment support increased the risk of back pain) and six reported an effect whereby lower levels of employment support increased the risk of back pain (Table 1). Of those six findings, three were judged as weak associations, one of moderate strength and two judged as strong effects.

### Co-worker support (CWS)

Seven studies were included within this analysis, six of those studies reporting no effect (Andersen et al. 2007; Hoogendoorn et al. 2001; Ijzelenberg and Burdorf 2005; Kaila-Kangas et al. 2004; Krause et al. 1998; Rugulies and Krause 2005) and one study reporting a reverse effect of higher CWS increasing the risk of LBP (Kerr et al. 2001). Examination of the studies revealed that six utilised an adequate measure of employment support with one study using a measure judged as poor (Ijzelenberg and Burdorf 2005). Included studies covered a range of geographical areas, had a broad selection of employment type, and a broad range of assessments for back pain. All studies used multivariate statistical testing, report an average level of response to follow-up at 77 %, had a mean follow-up period of 7.6 years, and all included samples of 500 participants or over.

### Supervisor support (SS)

Six studies were included within this analysis. Four studies reported no effect of SS on risk of LBP (Andersen et al. 2007; Hoogendoorn et al. 2001; Krause et al. 1998; Rugulies and Krause 2005) with two studies reporting a strong effect of lower levels of SS increasing the risk of LBP (Ijzelenberg and Burdorf 2005; Kaila-Kangas et al. 2004). Comparing studies that report no effect with those that do report an effect, all those reporting no effect were judged as having an adequate measure of SS, whereas one study reporting an effect (Ijzelenberg and Burdorf 2005) was judged as poor, using only a single question to assess support. Assessment of back pain was similar across all studies. Studies were also relatively similar on their geographic populations. All of the studies had sample sizes above 500. Average baseline response rates for studies reporting no effect was 75 % compared to 86 % for the Ijzelenberg and Burdorf (2005) study (Kaila-Kangas et al. 2004, failed to report a baseline response). Average attrition rates at follow-up for studies reporting no effect were 88 % compared to 57 % for the two studies that report an effect. However, this value of 57 % was markedly reduced by the Kaila-Kangas et al. (2004) study who report loss to follow-up at 33 % with the Ijzelenberg and Burdorf (2005) study reporting 86 %. The average follow-up time for studies that report no effect was 4.4 years in comparison with the studies that reported an effect were highly variable, with Ijzelenberg and Burdorf (2005) at 6 months and Kaila-Kangas et al. (2004) at 28 years.

### General work support (GWS)

In total, 13 studies report on 14 findings for risk of back pain and GWS. Overall, 10 studies (Clays et al. 2007; Elfering et al. 2002; Fransen et al. 2002; Ghaffari et al. 2008; Gheldof et al. 2006; Gonge et al. 2002; Harkness et al. 2003; Josephson and Vingard 1998; Larsman and Hanse 2009; Shannon et al. 2001) report no effect and 4 show an effect, of those 3 show a weak effect (Clays et al. 2007; Feuerstein et al. 2001; Leino and Hanninen 1995) and 1 reports a moderate effect (Stevenson et al. 2001). Studies reporting no effect all included an adequate assessment of GWS, whereas two studies reporting an effect (Feuerstein et al., Stevenson et al.) were judged to have poor assessments. Assessment of pain was variable in studies that did not report an effect with measurements of back pain measured via compensation claim records, current pain, pain in the previous week, or pain in the previous 12 months. The assessment was less variable for studies reporting effects, all of them assessing pain within the previous 6–12 months. Geographic locations are similar for studies. Employment type was similar between studies reporting an effect and those who did not. Average sample sizes were found to be similar. There are differences in the average baseline response with an average of 67 % for studies reporting no effect compared to 44 % for those reporting an effect but average attrition rates are similar. All studies employed multivariable analysis. The average follow-up time was 2.3 years (3 months to 6 years) for studies reporting no effect compared to 6 years (2–10 years) for studies that do report an effect.

### Employment social support and recovery from back pain

In total, 13 studies report 19 findings on the association between work support and return to work (RTW) for those with back pain. Overall, 11 findings report no association, 7 findings report associations whereby lower levels of work support delay RTW or recovery status and 1 study reports a weak reverse effect (Table 1). Of the findings of effect supporting an association between low work support and delays in RTW, 4 were judged as weak, 1 as moderate and 2 of strong effect.

### Co-worker support (CWS)

In total, 4 studies report effects, 2 finding an association that lower levels of CWS delay RTW status (Mielenz et al. 2008; van den Heuvel et al. 2004), 1 reporting a reverse effect (Schultz et al. 2004) and 1 reporting no association (Helmhout et al. 2010). All studies were judged to have used an adequate measure of CWS. The assessment of LBP varied between studies: the study finding no association (Helmhout et al. 2010) using recurring LBP in the previous 4 weeks, the study reporting a reverse effect (Schultz et al.) measuring pain and disability in the previous 6 months, and the 2 studies reporting a positive association using biomechanical assessment (Mielenz et al. 2008) and presence of LBP in the previous 12 months (van den Heuvel et al. 2004). Geographic locations were similar for all studies. The 2 studies that report an association drew their samples from general workers, whereas the study reporting no association used a military sample, and the study reporting a reverse effect recruited general workers on current compensation for their LBP. Average sample size was larger for the studies reporting an association (1,042 vs. 190), and they also report a greater average response rate (88 vs. 32 %). Average follow-up response rates were lower for the 2 studies reporting an association (69 %) compared to 85 % for the Schultz et al. (2004) study; Helmhout et al. (2010) failed to report on attrition. Multivariable statistical testing was used by studies reporting an association, the study who reported no association and the study who found a reverse effect both used univariable analysis. Follow-up time was variable with the studies reporting a positive effect having the largest difference (8 weeks and 3 years), the study reporting no association at 6 months and the study reporting the reverse effect at 3 months.

### Supervisor support (SS)

In total, 3 studies were included within this category. All studies reported no association between the level of SS and RTW status. All studies were judged to have adequate measures of SS, included a broad assessment of LBP, and covered a broad geographical area (Europe and USA). Multivariable testing was used by 2 studies (Mielenz et al. 2008; van den Heuvel et al. 2004). Length of follow-up was variable between studies with an average baseline response of 65 % and an average 68 % follow-up rate.

### General work support (GWS)

For the effects of GWS on RTW status, 9 studies (Dionne et al. 2007; Gheldof et al. 2006; Heymans et al. 2006; Karlsson et al. 2010; Lotters and Burdorf 2006; Morken et al. 2003; Soucy et al. 2006; Tubach et al. 2002; van der Giezen et al. 2000) report on 12 findings. Of those findings, 5 are of an association between lower levels of GWS and delays in RTW status (4 of weak effect and 1 strong) and 7 findings of no association. All but one study that report no association (Lotters and Burdorf 2006), and all but one study that report an association (van der Giezen et al. 2000) included measures of GWS judged to be adequate. Assessment of LBP is variable within studies that report an association and those that do not, including current pain at time of assessment to pain within the previous 5 years, consultations and ICD coding. Geographic locations are generally similar between studies. Recruitment samples for studies that report associations are from general and industry workers, and also those involved in compensation claims; for studies reporting no association, there is recruitment from industrial workers but also those who have indicated working status from a random population sample, and health care consulters where work type was not recorded. Average sample sizes, baseline response rates, follow-up rates and follow-up time were similar for studies reporting no association and those reporting associations. All studies, except van der Giezen et al. (2000) who reported an association, used multivariable analysis.

## Discussion

This review has carried out a systematic search for articles that reported on the effects of work social support on back pain from risk of occurrence and prognosis (recovery and return to work) studies. Overall, the evidence suggests no effect of work support as a risk factor for back pain; however, by examining the different types of support some distinctions occur. A similar picture emerges on the data and evidence for recovery and return to work with some evidence of CWS influencing outcome and mixed findings for GWS. The results suggest that employment-related support is less likely a factor on why someone gets back pain but could be an important factor on recovery and return to work once back pain is experienced.

### Risk of occurrence of back pain

The evidence suggests that the level of support perceived from co-workers is not a factor in risk of back pain. For CWS, the included studies, all showing no or a reverse effect, incorporated an adequate range of measures on CWS, a broad range of employment types and a broad assessment of back pain. The results for the effects of SS do show some effect is present. However, the studies reporting effects had less adequate assessments of SS and highly variable follow-up periods (6 months and 28 years) and so the effect, although strong in both studies, has to be tempered with these differences. More research is needed to investigate whether SS is a risk factor for back pain. The results on risk and GWS show a similar pattern with no or little effect and no discernible differences on the key extracted data between studies that reported an effect and those that did not. One exception to this is the lesser variability on the assessment of pain in studies reporting an effect (presence of back pain in the previous 6–12 months). This may have led to an inflated incidence rate compared to perhaps more stringent assessments of compensation claims or current pain used in some of the studies reporting no effect. However, notably three studies that reported no effect (Gheldof et al. 2006; Josephson and Vingard 1998; Larsman and Hanse 2009) could be considered as non-significant trends and so more information is needed before conclusions can be drawn.

### Prognosis for back pain

Overall, the evidence for prognosis is less clear with mixed findings for both CWS and GWS. The results for CWS, considering the key elements of study bias, suggest that the findings of an effect (less CWS delays recovery and return to work status) are more robust than those reporting no effect or a reverse effect. It may be that a supportive co-worker environment is important for those who have back pain, and this study’s finding supports the finding of a previous review (Steenstra et al. 2005), who showed a small pooled effect of CWS and work-related prognostic outcomes for those with back pain. The results for SS show no effect for all the included studies. This suggests that the perception of support directly from supervisors is not a factor in recovery. However, due to only three included studies, more research is needed. Findings are mixed for evidence of an effect of GWS on recovery and return to work with no apparent differences in key areas of bias between studies reporting and not reporting an effect. A reason for the stronger presence of an effect for GWS compared to SS could be that the measure of GWS is more than just a measure of support *per se*. For example, many of the studies that have measured general work support have included within their support measures aspects such as: perceived satisfaction of support (Leino and Hanninen 1995; Fransen et al. 2002), emotional aspects of support (Elfering et al. 2002), questions on work output (Fransen et al. 2002), conflict within the employment context (Larsman and Hanse 2009) or have used generic unspecified measures of support (Gonge et al. 2002; Ghaffari et al. 2008; Shannon et al. 2001;Morken et al. 2003; van der Giezen et al. 2000; Heymans et al. 2006). These aspects could be seen as support items but also as part of a larger concept of the workers’ general evaluation of their job. According to Karasek et al. (1998), aspects such as satisfaction with work, level of demands on the worker, the level of control the worker has, level of conflict at work are all important in their own right. It may be that the measures of general work support have been influenced by some of these factors. This therefore suggests that aspects involved in the supportive context for workers are important as prognostic factors for back pain; however, due to the variation in measurements used by studies in this review, the exact constructs relating to this are indistinct.

Taken together, the results for risk and prognosis show a weak effect of employment-related support for those with back pain. Less clear are the mechanisms that explain this association and this may be partly due to the ambiguity on what is meant by ‘support’ in an employment context. For example, a recent review by Woods (2005) included aspects of support such as satisfaction with employment, emotional support, conflict in the workplace, policy on occupational health, level of communication, health and safety policy, sickness absence policy, whereas other reviews such as Hartvigsen et al. (2004) have only reported on effects of direct co-worker support and supervisor support; Steenstra et al. (2005) and Hoogendoorn et al. (2001) have both included measures of problematic relations with other workers, whereas Kuijer et al. (2006) did not clearly specify what they meant by employment social support. This then broadens the scope of the concept of ‘support’ and this variation in definition may have contributed to the level of inconsistency described in previous reviews. Interestingly, this review could be construed as spanning this inconsistency, with no or very weak evidence of an effect for specific measures of CWS and SS (e.g. similar to Harvigsen et al.) but an increase in association for the generic GWS concept (e.g. similar to Woods). Many of the studies within the review who report GWS have combined measures of CWS and SS, and it is suggestive that some effect is there but it appears greater than the sum of its parts. Future research needs to consider the inherent complexity in the conceptualisation of employment social support (for a fuller explanation see “Appendix 4”). Furthermore, as mentioned in the introduction, the concept of employment co-worker and supervisor support forms only part of a larger model proposed by Karasek et al. (1998). There is a need to consider the component influence of employment social support as a moderator by using more sophisticated statistical modelling (e.g. path analysis, structural equation modelling) if we are to understand the role of employment social support, and in what context, on outcomes of spinal pain. Added to this is the evidence of the heterogeneity in the measurement of the outcome of back pain within this review. Studies differed in their assessment (patient rated, biomechanical testing, compensation status, different time scales for assessment) which makes comparisons all the more complex; future reviews should consider this issue.

### Comparison with other reviews

This review has concentrated on the effects of employment social support, whereas most other reviews have considered this as part of a wider search of employment psychosocial factors. This has led other reviews to include only a small number of studies on which to base their conclusions, for example, Steenstra et al. (2005) based theirs on four studies, Hoogendoorn et al. (2000) on six studies and Hartvigsen et al. (2004) on nine studies. The greater number of studies included in this review (thirty-two) has enabled a more specified focus on employment support type and outcome (risk and prognosis), which we believe has overcome some of the issues of heterogeneity and inconsistency described by previous reviews.

### Strengths and limitations

While this review has a comprehensive systematic search strategy, it did not include studies in languages other than English and so may have missed important findings; however, we did include studies from a range of countries worldwide. In addition, no review is completely immune from publication bias, and it may be the case that there are other findings (grey literature) we have not accessed. Strengths of the study are: the use of a systematic critical synthesis of the evidence which has enabled a closer inspection of the term employment social support and a better assessment of the types of support combined with an examination of individual study bias on the associations.

### Further research

This review has highlighted a need for consensus on what is meant by the term ‘employment social support’. As mentioned previously, there are a number of differing conceptualisations and future research needs to report on those concepts to facilitate easier comparisons for future reviews but also, more importantly, to understand what factors of employment social support associate with outcomes. Secondly, and related to the first point, there is a need for research to consider the role of theoretical models within their research. Many studies (over 50 % in this review) employed the Karasek Job Content Questionnaire, or a derivative, as their measure of employment social support. However, studies did not perform the appropriate analysis techniques to ascertain whether employment social support is a moderator component as prescribed by the Karasek model.

## Conclusion

This review has shown that employment-related support has little to no effect on risk of occurrence but a more notable effect on prognosis for those with back pain. The overall effect is weak for these findings; however, a greater understanding of the construct of ‘support’ in an employment context, and what factors interact with support, may lead to important pathways to reduce risk and reduce sickness absence, which could then be implemented by employers and occupational health practitioners.

## Appendix 1

See Table 2.

**Table 2:** Systematic review database search terms

Term	Major heading	Keywords	Search text
Medline			
Back pain	Back pain (exploded) Low back pain (exploded) Sciatica (exploded)	Back pain, backache, low back pain	(“Back pain”[Mesh] OR “Low back pain”[Mesh] OR “Back pain”[Text Word] OR “Backache”[Text Word]
Social support	Social support (exploded) Social isolation (exploded)	Interpersonal relations, interpersonal relation, social interaction, interaction social, social interactions, interactions social, employee health services, occupational health services, employment support, employment based support	(“Social support”[Mesh] OR “Social Isolation”[Mesh]] OR “Interpersonal relations”[Text Word] OR “Social interaction”[Text Word] OR “Social interactions”[Text Word] OR “interaction social”[Text Word] OR “interactions social”[Text Word] OR “employee health services”[Text Word] OR “occupational health services”[Text Word] OR “employment support”[Text Word] OR “employment based support”[Text Word])
Study setting	Cohort studies (exploded) Epidemiologic studies (exploded) Follow up studies (exploded) Prospective studies (exploded) Longitudinal studies (exploded) Cross-sectional studies (exploded) Health surveys (exploded)		(“Cohort studies”[Mesh] OR “Epidemiologic studies”[Mesh] OR “Follow up studies”[Mesh] OR “Prospective studies”[Mesh] OR “Longitudinal studies”[Mesh] OR “Cross sectional studies”[Mesh] OR “Health surveys”[Mesh])
AMED, IBSS and the British nursing index			
Back pain			DE “Back pain” or KW “Low back pain” or AB “Back pain” or AB “Low back pain” or AB “Lower back pain” or AB “Lumbago” or AB “Backache” or AB “Back ache” or AB “Lower back ache”
Social support			DE “Social support” or DE “Social networks” or DE “Friendship” or DE “Social interaction” or KW “Social support” or KW “Social networks” or AB “Social support” or AB “Social networks” or DE “Employment” OR DE “Employment support”
Study setting			(DE “Between groups Design” or DE “Cohort analysis” or DE “Followup studies” or DE “Longitudinal studies” or DE “Repeated measures” or DE “Quantitative methods” or DE “Mail surveys” or DE “Telephone surveys”) or (TX “Between groups design” or TX “Cohort analysis” or TX “Followup studies” or TX “Longitudinal studies” or TX “Repeated measures” or TX “Quantitative methods” or TX “Mail surveys” or TX “Telephone surveys”)
PsychINFO			
Back pain	Back pain (exploded)	Low back pain, backache, lumbago, back ache, lower back pains, low back ache	DE “Back pain” or KW “Low back pain” or AB “Back pain” or AB “Low back pain” or AB “Lower back pain” or AB “Lumbago” or AB “Backache” or AB “Back ache” or AB “Lower back ache”
Social support	Social support (exploded) Social networks (exploded) Social interaction (exploded)	Social support, social networks, work, employment	DE “Social support” or DE “Social networks” or DE “Social interaction” or KW “Social support” or KW “Social networks” or AB “Social support” or AB “Social networks” or DE “Job satisfaction” or DE “Employment status” or DE “Work attitudes toward”
Study setting	Between groups design (exploded or text terms) Cohort analysis (exploded or text terms) Followup studies (exploded or text terms) Mail surveys (exploded or text terms) Telephone surveys (exploded or text terms) Longitudinal studies (exploded or text terms)		(DE “Between groups design” or DE “Cohort analysis” or DE “Followup studies” or DE “Longitudinal studies” or DE “Repeated measures” or DE “Quantitative methods” or DE “Mail surveys” or DE “Telephone surveys”) or (TX “Between groups design” or TX “Cohort analysis” or TX “Followup studies” or TX “Longitudinal studies” or TX “Repeated measures” or TX “Quantitative methods” or TX “Mail surveys” or TX “Telephone surveys”)
EMBASE			
Back pain	Backache (exploded)		(Back AND Pain OR Back AND Injuries OR Back AND Pain AND with AND Radiation OR Back AND Pain AND without AND Radiation OR Backache OR Low AND Back AND Pain OR Low AND Back AND Ache OR Low AND Backache OR Mechanical AND Low AND Back AND Pain OR Recurrent AND Low AND Back AND Pain OR Postural AND Low AND Back AND Pain OR Lumbago OR Lumbalgesia OR Lumbal AND Pain OR Lumbar AND Pain OR Lumbalgia OR Lumbosacral AND Pain).ti,ab OR (exp BACKACHE/)
Social support	Social support (exploded) Social network (exploded) Social structure (exploded) Social interaction (exploded)		(exp *SOCIAL NETWORK/OR exp SOCIAL STRUCTURE/OR exp SOCIAL SUPPORT/OR exp SOCIAL INTERACTION/)
Study setting	Longitudinal study (exploded) Follow up study (exploded) Case–control study (exploded) Cross-sectional study (exploded) Cohort analysis (exploded) Epidemiology (exploded) Prevalence (exploded) Questionnaire (exploded)		(exp LONGITUDINAL STUDY/OR exp FOLLOW UP/OR exp CASE CONTROL STUDY/OR exp CROSS-SECTIONAL STUDY/OR exp COHORT ANALYSIS/OR exp EPIDEMIOLOGY/OR exp PREVALENCE/OR exp QUESTIONNAIRE/)
CINAHL			
Back pain	Back pain low back pain, sciatica	Back pain, low back pain, backache, back ache, lumbago	(MH “Back pain+”) or (MH “Lower back pain”) or (MH “Back”) or (“Lumbago”) or (“Lower back pain”) or (“Back pain”) or (“Neck pain”) or (“Backache”) or (“Back ache”)
Social support	Social support Norbeck social support questionnaire Social support Iowa NOC Social support index Support, psychosocial (exploded) Social networks	Social support Social networks	(“Social support”) or (Work psychosocial factors) or (MH “Norbeck Social support Questionnaire”) or (MH “Social support (Iowa NOC)”) or (MH “Social support index”) or (MH “Support, Psychosocial+”) or (“Social networks”) or (MH “Social networks”)
Study setting	Experimental studies Nonexperimental studies Concurrent prospective studies Cross-sectional studies Health policy studies		(MH “Experimental studies”) or (MH “Nonexperimental studies”) or (MH “Concurrent prospective studies”) or (MH “Cross sectional studies”) or (MH “Health policy studies”)

## Appendix 2

See Table 3.

**Table 3:** Quality assessment

First author	Scoring criteria for quality assessment															
	All designs											Cohort			Case control	
	1	2	3	4	5	6	7	8	9	10	11	12	13	14	15	16
Andersen	Y	Y	Y	Y	Y	Y	Y	Y	Y	Y	Y	Y	Y	Y		
Clays	Y	Y	N	Y	Y	Y	Y	Y	Y	Y	N/R	Y	N	Y		
Dionne	Y	Y	Y	Y	Y	Y	Y	Y	Y	Y	Y	N	Y	Y		
Elfering	Y	N/R	N	N	N	Y	Y	Y	N	Y	Y	Y	Y	Y		
Feuerstein	Y	Y	Y	Y	Y	N	Y	Y	Y	Y	N	N/A	N/A	N/A	Y	Y
Fransen	Y	Y	N	N	N	Y	Y	Y	Y	Y	N	N/R	N/R	N		
Ghaffari	Y	N	N	Y	N	Y	Y	Y	Y	N	Y	N/R	Y	Y		
Gheldof	Y	Y	Y	Y	Y	Y	Y	Y	Y	Y	N	Y	N	Y		
Gonge	Y	Y	N	N	Y	Y	Y	Y	Y	Y	Y	N	Y	Y		
Harkness	Y	N	N	N	Y	Y	Y	Y	Y	N	Y	Y	N	Y		
Helmhout	Y	Y	Y	Y	N	Y	Y	Y	Y	Y	N/R	N/R	Y	Y		
Heymans	Y	Y	Y	Y	N/R	Y	Y	Y	Y	N	Y	Y	Y	Y		
Hoogendoorn	Y	Y	Y	N	N	Y	Y	Y	Y	Y	Y	N	N	Y		
Ijzelenberg	Y	Y	Y	Y	N	N	Y	Y	Y	N	Y	Y	Y	Y		
Josephson	Y	Y	Y	N	N	Y	Y	Y	Y	Y	N	N/A	N/A	N/A	Y	Y
Kaila-Kangas	Y	Y	N	Y	N	Y	Y	Y	Y	Y	N/R	N/R	N/R	Y		
Karlsson	Y	Y	Y	Y	N/R	Y	Y	Y	Y	Y	Y	N/R	N	Y		
Kerr	Y	Y	Y	Y	Y	Y	Y	Y	Y	Y	N	N/A	N/A	N/A	Y	Y
Krause	Y	Y	Y	N	Y	Y	Y	Y	Y	Y	Y	Y	N	Y		
Larsman	Y	Y	N/R	N	N	Y	Y	Y	Y	Y	Y	N/R	N	Y		
Leino	Y	Y	Y	N	Y	Y	Y	Y	Y	N	N	Y	N	Y		
Lotters	Y	Y	Y	Y	Y	N	Y	Y	Y	N	N	N	Y	Y		
Mielenz	Y	Y	N	Y	Y	Y	Y	Y	Y	Y	Y	N	Y	N		
Morken	Y	Y	N/R	Y	Y	Y	Y	Y	Y	Y	Y	N/R	N	Y		
Rugulies	Y	Y	Y	Y	Y	Y	Y	Y	Y	Y	N	Y	Y	Y		
Schultz	Y	Y	Y	Y	Y	Y	Y	Y	Y	Y	N	Y	Y	N		
Shannon	Y	Y	Y	Y	Y	N	Y	N	Y	Y	Y	Y	N	Y		
Soucy	Y	Y	Y	Y	Y	Y	Y	Y	Y	Y	N	N	N	Y		
Stevenson	Y	Y	Y	N	N/R	N	Y	Y	Y	N	N/R	N/R	N/R	Y		
Tubach	Y	Y	Y	Y	Y	Y	Y	Y	Y	N	Y	Y	N	Y		
van den Heuvel	Y	Y	Y	Y	N	Y	Y	Y	Y	Y	Y	N	N	Y		
van der Giezen	Y	Y	Y	Y	Y	N	Y	N	Y	Y	Y	Y	N	Y		
Overall total %	100	91	69	69	60	81	100	94	97	75	56	48	41	90	100	100

*Y* *yes, N* *no, N/R* *not recorded, N/A* *not applicable*

Quality assessment scoring key (*CC* Case control, *CH* Cohort)

1: Does the study have a clear defined research objective? CH/CC

2: Does the study clearly describe the recruitment procedure? CH/CC

3: Does the study adequately describe the inclusion/exclusion criteria? CH/CC

4: Does the study report on the population parameters and demographics? CH/CC

5: Does the study report participation rates and provide evidence of comparisons of responders and non-responders? CH/CC

6: Does the study include the sufficient assessment of social support? Criteria—Higher quality where measure is validated or measures at least two dimensions. CH/CC

7: Does the study adequately report on the strength of effect (e.g. ways of calculating effect size, reporting of confidence intervals)? CH/CC

8: Does the study use multivariate analysis? CH/CC

9: Is the study sample size appropriate for the analysis used? CH/CC

10: Do the authors report on the limitations of their study? CH/CC

11: Does the study report a participation rate at baseline >70 %?CH/CC

12: Does the study report attrition rates and provide evidence of comparisons of responders and non-responders? CH

13: Does the study report an attrition rate <20 %? CH

14: Does the study have a follow up time period >6 months? CH

15: Does the study use the same population for cases and controls? CC

16: Are the study controls adequately (e.g. no pain for >3 months) screened for symptoms compared to cases? CC

## Appendix 3

See Table 4.

**Table 4:** Data extraction tables for included studies

Author (years)	Country	Study population	Design	Main study focus	LBP assessment	Work support assessment	Findings	Results
Andersen et al. (2007)	Denmark	General workers sample	Prospective cohort with a 2 year follow up	Psychosocial risk factors for musculoskeletal symptoms within workers	Presence of pain in previous 12 months + absence from work	Danish National institute of Occupational health Questionnaire—CWS and SS	Low SS not a risk for LBP CWS as a non-significant risk factor for LBP	HR 1.1 (0.8–1.6) HR 1.1 (0.8–1.6)
Clays et al. (2007)	Belgium	General workers sample	Prospective cohort over 6 years	The impact of psychosocial factors on LBP	Nordic questionnaire >8 days in previous 12 months	Karasek Demand Control model—GWS	Low GWS increased risk of LBP in men No association between GWS and risk in women	RR 1.2 (1.02–1.42) RR 1.00 (0.8–1.24)
Dionne et al. (2007)	Canada	Consulters for LBP who have been absent from work for at least 1 day	Prospective BL, 6 week, 12 week, 1 year and 2 year follow ups	RTW for those with LBP	RMDQ, pain levels, fear avoidance	Work APGAR	No significant role for GWS on RTW	OR 4.76 (0.43, 52.13)
Elfering et al. (2002)	Switzerland	Workers (unspecified)	Prospective cohort over 5 years	Social support at work and risk of LBP	Nordic questionnaire, pain frequency and intensity, RMDQ, McGill Questionnaire	General questions on support in employment	No significant association between low GWS and LBP	N/S
Feuerstein et al. (2001)	USA	Military personnel	Case control	Workplace psychosocial factors associated with sickness absence due to LBP	Self report LBP symptoms, NIOSH survey. One episode of LBP in past 12 months resulting in an episode of sickness absence	Work environment scale (inclusive of one question on GWS)	Participants with low GWS were at higher odds of getting LBP	OR 1.22 (1.05, 1.36)
Fransen et al. (2002)	New Zealand	Workers claiming compensation	Prospective cohort 3 months	Risk factors associated with transition from acute to chronic LBP status	Work-related back injury codes on claim files and the Owestry disability index	Work APGAR—7 questions on perception of support at work (GWS)	Perceived levels of GWS support at work did not predict transition from acute to chronic status and was not included in the final multivariable model	N/S
Ghaffari et al. (2008)	Iran	Industrial workers	Prospective 1 year study	Psychosocial factors predictive of risk of LBP in workers	MUSIC measure—assesses the presence of aches and pain in lower back	GWS measure (unspecified)	No significant associations found for GWS and LBP	OR 0.5 (0.3, 1.0)
Gheldof et al. (2006)	Netherlands	Industrial workers	Prospective cohort 18 months	Risk and recovery from LBP in a work setting	Current pain intensity (NRS) pain radiation	Karasek Demand Control model—GWS	No significant associations found for GWS and risk of LBP No significant associations found for GWS and short term recovery No associations found for long term recovery	OR 1.19 (0.98, 1.44) OR 0.88 (0.72, 1.07) OR 0.97 (0.87, 1.07)
Gonge et al. (2002)	Denmark	Nursing personnel	Prospective cohort 6 months	Impact of psychosocial factors on LBP	Presence of LBP, pain intensity and pain over 3 months	Questions on the frequency of GWS	There was no association between GWS and LBP	OR 1.7 (0.7, 4.3)
Harkness et al. (2003)	UK	General workers sample	Prospective 1 year and 2 year study	Risk factors for new onset LBP in workers	Back pain presence in the past month for 1 day or longer	Karasek Demand Control model—GSW	No significant association found for GWS and risk of LBP	OR 1.4 (0.5–3.7)
Helmhout et al. (2010)	Netherlands	Military personnel	Prospective 6 months	Prognostic factors for clinical improvement for those with LBP	4 weeks of recurring LBP at least 3 times per week	Karasek Demand Control model—CWS and SS	No significant association of CWS and disability related to LBP No significant association of SS and disability	OR 0.88 (0.64, 1.21) OR 1.07 (0.82, 1.09)
Heymans et al. (2006)	Netherlands	General workers sample	Prospective 1 year study	Beliefs and expectations of those with LBP about RTW	Presence of LBP, RMDQ and RTW status	Karasek Demand Control model—GWS	Increased GWS was shown to increase RTW status for those with back pain	HR 1.04 (1.0, 1.08)
Hoogendoorn et al. (2001)	Netherlands	General workers sample	Prospective 3 year study	Psychosocial work factors and LBP	Nordic questionnaire. Regular or prolonged back pain in previous 12 months	Karasek Demand Control model—SS and CWS	There was no significant association between SS and risk of LBP There was no significant association between levels of CWS and risk of LBP	RR 1.30 (0.75, 2.26) RR 1.59 (0.89, 2.86)
Ijzelenberg and Burdorf (2005)	Denmark	Industrial workers	Prospective 6 month study	Work-related psychosocial factors and risk of MSK	Nordic questionnaire. MSK pain within previous 12 months (BL) and previous 6 months (FU)	Karasek Demand Control model—SS and CWS	Less SS was associated with increased risk of LBP Less CWS was not associated with increased risk of LBP	OR 2.06 (1.35, 3.14) OR 1.52 (0.97, 2.38)
Josephson and Vingard (1998)	USA	Nursing personnel	Case control	Workplace factors as risk for LBP in female nursing cohort	Clinical examination and referral for treatment for LBP	2 interview questions and 6 items within questionnaire on GWS	No significant association between low GWS and increased risk of LBP	OR 2.4 (0.9, 6.4)
Kaila-Kangas et al. (2004)	Finland	Metal industry workers (blue and white collar)	Prospective design linked to hospitalisation records 28 years	ICD codes for hospitalisation for back disorder	ICD codes	Karasek Demand Control model—SS and CWS	Significant association found for SS No significant association found for CWS	RR 3.28 (1.32–8.17) RR 1.08 (0.46–2.54)
Karlsson et al. (2010)	Sweden	Random population	Prospective cohort 2 years	Psychosocial factors related to sickness absence	Presence of LBP in past 5 years	Karasek Demand Control model with general question on support at work - GWS	No significant effect for general social support at work and sickness absence due to LBP	RR/OR 0.97 (0.088, 1.07)
Kerr et al. (2001)	Canada	Industrial workers	Case control	Psychosocial factors associated with LBP	Biomechanical assessment	Karasek Demand Control model—CWS	Higher levels of CWS increased risk of LBP	OR 1.6 (1.07, 2.32)
Krause et al. (1998)	USA	Transit operators	Prospective cohort 5 year study	Work-related risk factors of back injury	Compensation claims for back injury	Karasek Demand Control model—SS and CWS	No significant association found for CWS and spinal injury Non-significant trend reported for lower SS and risk of spinal injury	OR 1.00 (0.75, 1.35) OR 1.30 (0.99, 1.72)
Larsman and Hanse (2009)	Sweden	Female human service workers	Prospective cohort 18 month study	Impact of the demand control model on neck, shoulder and back pain in workers	Nordic questionnaire (MSK)	Karasek Demand Control model - GWS	No significant association found for GWS and back pain	OR 1.37 (0.97, 1.92)
Leino and Hanninen (1995)	Finland	General workers sample	Prospective cohort 10 year study	Psychosocial work factors and back pain	Presence of pain symptoms within previous 12 months	6 questions on social relationships at work	Significant association found between lower GWS support at work and greater risk of LBP	Beta (0.146), *p* = 0.001
Lotters and Burdorf (2006)	Netherlands	Workers registered on an occupational health register	Prospective cohort 1 year	Prognostic factors of sickness absence due to MSK	Consultation registry Nordic and RMDQ	Karasek Demand Control model—GWS	No significant effect for relation with colleagues and sickness absence in those with LBP	HR 1.05 (0.86–1.28)
Mielenz et al. (2008)	USA	General workers sample	Prospective 8 week study	Work-related psychosocial factors associated with LBP	Biomechanical assessment	Work APGAR—7 questions on CWS and SS	No association between levels of SS and recovery There was an increase in recovery rates for those with higher levels of CWS	RR 0.71 (0.34, 1.48) RR 1.55 (1.04, 2.34)
Morken et al. (2003)	Norway	Industrial workers	Prospective 2 year study	Long and short term sickness absence due to pain and LBP	Nordic questionnaire for presence of pain in previous 12 months	Karasek Demand Control model—GWS	No association between GWS and LBP for long term sickness absence Significant association found between low levels of GWS and increased short term sickness absence	RR 1.0 (0.8, 1.3) RR 1.28 (1.11, 1.49)
Rugulies and Krause (2005)	USA	Transit operators	Prospective cohort 7.5 year study	Job strain and incidence of LBP and neck pain	Worker compensation claims and ICD coding for back and neck disorders	Karasek Demand Control model—SS and CWS	No associations found for CWS with LBP No associations found for SS with LBP	HR 1.00 (0.78, 1.29) HR 1.02 (0.77, 1.34)
Schultz et al. (2004)	Canada	General workers sample (compensation claimants)	Prospective cohort study 3 month	Psychosocial factors predictive of LBP disability and RTW status	McGill pain questionnaire CPG	Karasek Demand Control model—CWS	Low levels of CWS predicted quicker RTW status	Beta 0.2, p = 0.079
Shannon et al. (2001)	Canada	Hospital workers	Prospective cohort 3 year study	Predictors of changes in MSK health	Presence and pain level of back pain in previous week	10 item measure of emotional and instrumental support at work GWS	GWS did not remain as a predictive factor of MSK status	N/S
Soucy et al. (2006)	Canada	General workers sample (compensation claimants)	Prospective cohort study 6 month	Work-related factors contributing to chronic disability in those with LBP	Pain intensity and RMDQ	8 item questionnaire on work social support GWS	Low GWS increased risk of chronic disability	OR 1.11 (1.02, 1.22)
Stevenson et al. (2001)	Canada	Industrial workers	Prospective cohort 2 year study	Risk of LBP	Self rate question on presence of LBP in previous 6 months. Mechanical lifting test	1 question on having a confidante at work GWS	Absence of confidante at work increased risk of LBP	Beta 0.27, OR 1.7, *p* = 0.039
Tubach et al. (2002)	France	Industrial workers	Prospective cohort 4 year study	Risk factors for sickness absence due to LBP	Nordic questionnaire for LBP	Karasek Demand Control model—GWS	Lower levels of GWS were shown to significantly increase sickness long term absence (> 8 days) There was no association between GWS and shorter term sickness absence	OR 3.4 (1.6, 7.3) OR 1.4 (0.9, 2.3).
van den Heuvel et al. (2004)	Netherlands	General workers sample	Prospective cohort 3 year	Sickness absence due to LBP	Nordic questionnaire, presence in previous 12 months, pain intensity and RMDQ	Karasek Demand Control model—SS and CWS	Significant effect found for low CWS and increased sickness absence No significant effect found for SS and sickness absence	OR 4.08 (1.59–10.05) OR 2.69 (0.85–8.44)
van der Giezen et al. (2000)	Netherlands	General workers	Prospective cohort 12 month study	Precdictors of RTW status for those sick listed (>90 days) due to LBP	ICD codes for back pain disorder and pain manikin	Karasek Demand Control model—GWS	Greater levels of GWS predicted a quicker RTW status	OR 1.16 (1.03, 1.30)

*LBP* low back pain, *RTW* return to work, *SS* Supervisor support, *CWS* co-worker support, *GWS* general work support, *N/S* not significant, *OR* odds ratio, *HR* hazard Ratio, *RR* relative risk

## Appendix 4: Assessment of employment social support

As evidenced from this review the assessment of employment support is multifaceted. Initially Johnson and Hall (1988) introduced the concept of work social support in the context of Karasek’s (1981) ‘Demand Control Model’ of job strain and illness outcomes. They showed that the level of social interaction between workers modified the association between job strain and cerebrovascular disease. Initial conceptualisation and measurement was restricted to a measure of the social interaction between workers with measurement of the level of communication between workers in times of work breaks, and as part of their working day in addition to the social interaction between workers outside of the employment context. Karasek et al. (1998) added to this concept by assessing the level of emotional support from both co-workers and supervisors as well as assessing the level of instrumental support (i.e. getting assistance to get their job done). The majority of the studies included within this review have based their assessment on the Karasek model, or the Work Apgar measure (Bigos et al. 1991); both of which primarily assess relationships between the worker and co-worker or supervisor, as well as the general work atmosphere. However Woods’ (2005) qualitative review acknowledged that other aspects of support may be equally important and included additional concepts such as; acceptance by peers at work, structural support (i.e. health and safety policy, management of occupational health), health specific (i.e. the ability to discuss health issues with employers), work and personal issues (the ability to discuss issues with employers both about work and personal), level of satisfaction, level of conflict and hostility within work, working alone and feeling isolated, social support outside of the work context.

This additional level of complexity is reflected within research on social support in general. Chronister et al. (2006) discusses the issue on the assessment of general social support and conceptualises the contingencies for social support on a number of differing levels. The first level is the structure; network (who offers the support), size (what size is the network, how many people), frequency (how frequent is the support available). The second level is support type; instrumental (actual practical support given by others), emotional (ability to discuss emotional issues), advice (having the availability to source advice specific to the issues the person faces), appraisal/affirmation (being affirmed and acknowledged by others). The third level is the perception of the support; it may be that all these levels of support are in place but the perception by the person will still be key as to whether the support is seen as helpful. For example, a study by Masters et al. (2007) considered social support within a health context and showed that social support can be perceived differently dependent on who is giving the support, over and above having the availability of the support.

The above evidence illustrates the complexity inherent when assessing employment social support. Future research of employment support needs to acknowledge and accommodate the complexity if we are to assess the estimates of the effect of employment social support on the outcomes for those with back pain.
